# Prevalence of Anti-JC Virus Antibody in Multiple Sclerosis Patients in Kuwait

**DOI:** 10.1155/2014/861091

**Published:** 2014-01-22

**Authors:** S. Lamdhade, A. Ashkanani, R. Alroughani

**Affiliations:** ^1^Division of Neurology, Department of Medicine, Amiri Hospital, Arabian Gulf Street, 13041 Kuwait City, Kuwait; ^2^Neurology Clinic, Dasman Diabetes Institute, P.O. Box 1180, 15462 Dasman, Kuwait

## Abstract

*Background*. Multiple sclerosis (MS) therapeutics entered a new era after the development of anti-JC virus (anti-JCV) antibody assay that assesses the risk of development of progressive multifocal leukoencephalopathy (PML) in patients treated with natalizumab. *Objective*. To determine the prevalence of anti-JCV antibody among MS patients in Kuwait. *Methods*. Using the national MS registry, demographics and disease characteristics of MS patients who were screened for anti-JC virus antibody were collected. The prevalence of anti-JCV antibody seropositivity and its association with demographic and disease characteristics were evaluated. *Results*. Out of 110 screened MS patients for anti-JCV antibodies, 65.5% were females. Mean age and disease duration were 29.23 ± 8.55 and 5.39 ± 5.04 years, respectively. 47.3% of patients were already on natalizumab and 52.7% of patients were screened for stratification to either natalizumab or a different Disease Modifying Therapy (DMT). The overall prevalence of anti-JC virus antibody was 40%. Gender (*P* = 0.69), disease duration (*P* = 0.11), and number of natalizumab infusions (*P* = 0.64) were not associated with seropositivity. Patients aged ≥30 years were more likely to be seropositive (*P* = 0.01). *Conclusion*. The prevalence of anti-JCV antibody is slightly lower than what is reported in published studies. Seropositivity was associated with an increasing age of MS patients.

## 1. Introduction

Progressive multifocal leukoencephalopathy (PML) is a rare neurological disease, which is caused by JC virus (JCV) infection. Reactivation of JCV occurs usually in immune-compromised or AIDS patients. JCV infection has gained importance to neurologists after the report of two PML cases in multiple sclerosis (MS) patients who received natalizumab [1].

The tremendous advances in the understanding of MS pathology and pharmacotherapy have improved the patients' outcomes significantly. Given the concern of PML, the benefit of natalizumab treatment is to be weighed against its risk. After the recent development of blood anti-JC virus antibody test, stratification of MS patients to various disease modifying therapies (DMTs) entered a new era [2]. Hence, studying the prevalence of JCV in MS patients became important, as it would impact the clinical decision-making, especially in patients who had received prior immunosuppressants or are receiving natalizumab for more than 24 months. Although many cases of PML have been reported from various parts of the globe, the relationship between the number of natalizumab infusions and risk of PML development has not been clearly established yet [3].

Few studies indicated that approximately 60–70% of MS patients were seropositive when screened for anti-JCV antibody [4]. Most of the available data come from North America and Europe. We have studied the prevalence of anti-JC virus antibody in Kuwait, which represents one of the highest regions of Middle East affected by MS [5].

## 2. Methods

We have retrospectively assessed the database of MS patients who were screened for anti-JCV antibody using the MS registry in two MS centers in Kuwait in the period between October 2012 and June 2013. Patients who were diagnosed with MS according to the revised 2010 McDonald criteria [6] were included while patients with other demyelinating disorders such as neuromyelitis optica (NMO) or acute disseminated encephalomyelitis (ADEM) were excluded. Demographics, clinical and treatment characteristics, and the indications of anti-JCV antibody testing were collected. The STRATIFY JCV antibody test is an enzyme-linked immunosorbent assay (ELISA) [7]. A two-step assay to determine anti-JCV antibodies was applied to the samples performed at Focus Diagnostics (Cypress, CA, USA) and sponsored by Biogen Idec (Cambridge, Massachusetts, USA) [7]. Samples with low-level reactivity in the detection assay were retested in a confirmatory (inhibition) assay to confirm presence or absence of JCV-specific antibodies. The main outcome measure was to determine the prevalence rate of anti-JCV antibody in MS patients. Furthermore, the association between seropositivity of anti-JCV antibody and prespecified demographics and disease characteristics was assessed. Several factors such as age, gender, disease duration, mean expanded disability status scale (EDSS) score at time of anti-JCV antibody testing, number of natalizumab infusions, and prior immunotherapy were studied. Statistical analysis was done using SPSS version 21.0 to calculate the significance through ANOVA and chi-square tests.

## 3. Results

Of 110 screened MS patients for anti-JC virus antibodies, 72 (65.5%) were females. Patients' demographics, clinical characteristics, and their current medications were listed in Table 1. The mean age and disease durations were 29.23 ± 8.55 years and 5.39 ± 5.04 years, respectively. Most of the patients (89.09%) had relapsing remitting MS and the mean EDSS score of the studied cohort was 3.75 ± 1.45 as shown in Table 1. The indication for screening was either to test patients who were already on natalizumab (47.3%, *n* = 52) or to stratify patients to be treated with either natalizumab or other disease modifying therapy (52.7%, *n* = 68).

The overall prevalence of seropositivity to anti-JC virus antibody was 40% (*n* = 44), of whom 68.2% were females. The characteristics of seropositive patients are shown in Table 2. Clinical parameters such as gender (*P* = 0.69), disease duration (*P* = 0.11), and the number of natalizumab infusions (*P* = 0.64) were not associated with seropositivity. Patients aged ≥ 30 years were more likely to be seropositive (*P* = 0.011) as outlined in Table 3.

After the results of anti-JCV antibody in patients who were not on natalizumab, 7 seropositive patients were prescribed natalizumab while 15 and 3 seropositive patients were prescribed fingolimod and interferons, respectively. Among seropositive patients who were tested while on natalizumab, 9 patients had subsequently stopped natalizumab (mean infusions 20; range 12–26) while 10 patients continued natalizumab (mean infusions 16.9; range 8–33). The mean infusion was 17.63 ± 10.48 for all patients who continued natalizumab irrespective of anti-JCV serostatus. There was no association between prior exposure to DMTs and anti-JCV antibody seropositivity in patients who were already on natalizumab as shown in Table 4. There were no reported cases of PML in Kuwait till date.

## 4. Discussion 

There is limited data on prevalence of anti-JCV antibody in the general population. A European study indicated that anti-JCV seropositivity was 58% in 20–29-year age group and increased to 68% in 50–59-year age group [8]. A similar incremental trend was observed in a longitudinal Australian population study as anti-JCV seropositivity was 60% in people younger than 50 years, 68% in 50–60-year age group, and 64% in people older than 70 years [9].

The status of anti-JCV antibody is important in assessing the risk of PML in MS patients where certain DMTs may be implicated. Natalizumab can reactivate JCV in the CNS but its mechanism is not clear [10]. Although infection by JC virus is a prerequisite for PML, the mechanism by which natalizumab can react with JCV in the CNS is not clear. Other factors such as prior immunosuppression and duration of natalizumab treatment may potentially increase the risk [11].

In MS cohorts, the prevalence of anti-JCV antibody varies across the geographical regions. A large multicenter study conducted in nine countries showed an overall prevalence of 57.6% [4]. Gorelik et al. observed higher prevalence of anti-JCV antibody in Europe and North America compared to Australia and New Zealand [12]. The false-negative rate of the ELISA was calculated to be approximately 2.5%, with an upper 1-sided confidence limit of 4.4% [12].

The prevalence of anti-JCV antibody in our cohort was lower than most of the published data. The small number of patients and the younger cohort could explain this. All the blood samples were sent to a central laboratory (Focus Diagnostics, Cypress, CA, USA), which had been used by other studies reporting the results of anti-JCV antibody prevalence rates. Hence, the sensitivity of the test did not have a significant impact on the overall results.

Similar to other studies, the seropositivity increased with the age in our cohort. The mean age was higher in seropositive group (33.0 versus 29.2 years; *P* = 0.023). Higher seropositive in men was common but not universally seen which could be due to higher age at onset in men [1, 3]. Gender did not seem to influence our results despite the observed non-significant predominant seropositivity in females (68.18%). Similarly, other studies did not find any significant gender difference [13, 14], suggesting that a larger sample size might be needed to better assess the gender difference.

A recent multinational study suggested that prior DMD was not an important factor for higher rates of seropositivity [4]. Similarly, Miller et al. did not find any correlation when first-line DMTs were used prior to the screening test in 239 patients [15]. Furthermore, there was no specific pattern between the type or the duration of prior immunosuppressant therapy and anti-JCV antibody seropositivity [16].

As far as number of natalizumab infusions was concerned, Bozic et al. did not find an increase in seropositivity when assessing 12-month increment of infusions [2]. In a study using JCV DNA in plasma with a mean infusion of 22 months, no significant correlation was observed with the number of natalizumab infusions [17].

Our study had several limitations. First, it included small number of patients, yet it represented the first regional cohort that would help in establishing a registry of anti-JCV antibody in the region. Second, there was a referral bias since most of the referrals to our MS centers were young MS patients with short disease duration. This could have impacted the overall prevalence of anti-JCV since age was a significant independent factor. Larger longitudinal studies are needed to clarify the interplay of multiple putative risk factors for proper risk stratification of PML.

In summary, the prevalence of anti-JCV antibody in MS cohort in Kuwait was 40%. This was slightly lower than European and North American data. Except for increasing age, we did not find any significant correlations between the presence of seropositive anti-JCV antibody and gender, prior first-line or immunosuppressant exposure, or number of natalizumab infusions. Although our cohort represents the first report from the Middle East, it underscores the urgent need of population-based seroprevalence determination in both healthy and MS cohorts.

## Figures and Tables

**Table 1:** Clinical characteristics of cohort (*n* = 110).

Characteristics	*N*, SD (%)
Mean age (years)	29.23 ± 8.55
Gender	
Female	72 (65.5%)
Male	38 (34.5%)
Mean disease duration (years)	5.39 ± 5.04
MS course	
Relapsing remitting	98 (89.09%)
Secondary progressive	12 (10.91%)
Mean EDSS score	3.75 ± 1.45
Current DMTs when tested	
None	11 (10%)
IFN beta 1a IM	18 (16.36%)
IFN beta 1a SC	10 (9.09%)
IFN beta 1b SC	13 (11.82%)
Fingolimod	5 (4.55)
Natalizumab	52 (47.27%)
Others	1 (0.91%)

**Table 2:** Characteristics of anti-JCV antibody seropositive group (*n* = 44).

Characteristics	*N*, SD, (%)
Mean age (years)	33 ± 7.84
Gender	
Female	30 (68.2%)
Male	14 (31.8%)
Mean disease duration (years)	6.95 ± 6.67
MS course	
Relapsing remitting	38 (83.4%)
Secondary progressive	6 (13.6%)
Mean EDSS score	3.88 ± 1.53
Current DMTs when tested	
None	4 (9.1%)
Other DMTs except natalizumab	23 (52.2%)
Natalizumab	17 (38.6%)

**Table 3:** Analysis of the status of anti-JCV antibody according to prespecified demographics and disease characteristics.

Variables	Anti-JCV antibody status		*P* value
	Negative	Positive	
Gender			
Female	42	30	0.69
Male	24	14	
Mean age	29.23 ± 8.72	33.0 ± 7.81	
<30 years	36	13	0.02*
≥30 years	30	31	0.01*
Mean disease duration (years)	5.39 ± 4.52	6.95 ± 5.66	0.11
Mean EDSS score	3.67 ± 1.41	3.88 ± 1.53	0.28

*Statistically significant.

**Table 4:** Analysis of demographics and disease characteristics in patients who were already on natalizumab (*n* = 52).

Variables	Anti-JCV antibody status		*P* value
	Negative (*n* = 33)	Positive (*n* = 19)	
Gender			
Female	17	13	0.26
Male	16	6	
Mean age (years)	28.09 ± 8.58	31.95 ± 6.08	0.09
Mean MS duration (years)	5.36 ± 3.93	5.89 ± 3.46	0.63
Mean EDSS	3.53 ± 1.42	3.53 ± 1.51	0.42
Mean number of natalizumab infusions	18.42 ± 12.32	19 ± 17.63	0.64
Prior exposure to *DMTs			
Yes	7	3	0.73
No	26	16	

*DMTs: disease modifying therapies.
